# Recent Observations on the Treatment of Gonorrhea

**Published:** 1901-02

**Authors:** Thomas C. Leitch

**Affiliations:** Brooklyn, N. Y.


### RECENT OBSERVATIONS ON THE TREATMENT OF GONORRHEA.

BY THOMAS C. LEITCH, M.D.,

BROOKLYN, N. Y.

There is a passage in Juvenal in which allusion is made to a disgusting disorder which appears to resemble venereal disease. Celsus prefaces what he says on the subject with an apology, and then proceedes to speak of the disease which seems to bear a striking analogy to the modern phimosis of gonorrhea. Persons afflicted with these maladies prayed to Juno for relief, and used the aster atticus by way of medicine. While the description of the disease, therefore, appeared quite early in the history of medicine, its treatment has been but recently placed upon a scientific foundation. In fact, until the discovery of the gonococcus by Neisser the treatment was entirely empirical. The discharge being considered as a manifestation of a catarrhal inflammation of the urethra, the chief therapy consisted in the administration of internal antiblennorrhagics, such as cubebs, copaiba, buchu, and the local injection of astringents. This method has not yet entirely fallen into disuse, although there is no doubt that much damage has been inflicted by the indiscriminate administration of large doses of uro-genital remedies, both by giving rise to gastric troubles and irritation and inflammation of the kidneys. To radically cure a case of gonorrhea it is now recognized that measures must be adopted for the destruction of the exciting cause, namely, the gonococcus. To accomplish this I have derived the most satisfaction from the use of protargol, a new remedy to which attention was first directed several years ago by Professor Neisser.

Protargol, although a silver preparation, differs entirely from nitrate of silver, which has always been esteemed one of the most valuable remedies in the treatment of gonorrhea. Unlike the latter, it is not affected by sodium chloride or albumen, two subtances which are always present in gonorrheal discharges. It is a very stable compound, and solutions may be prepared either with distilled or hydrant water, as I have not been able to notice any difference in this repect, although the nitrate is readily precipitated by the presence of salts in the water. This is readily shown by a simple experiment: if nitrate of silver be added to urine a chloride is quickly formed, while the addition of protargol does not lead to any changes.

Judging from my own experience there are no agents which will afford as much relief in urethral affections as the silver compounds. The nitrate would be an excellent remedy were it not for its caustic action. I have seen this caustic effect last as long as two days in some instances, although in others it passed off within an hour. Protargol has all the good qualities of the nitrate and none of its irritating effects, and hence I now use it exclusively in place of the former salt. It affords as much relief, and its use is unattended with the pain and burning so common with the nitrate.

In acute gonorrhea protargol produces a permanent cure in a short time. For the past two years I have used the Valentine irrigation method, and have tried all kinds of solutions, from bichloride of mercury to carbolic acid, but discarded one after another. The last one that I used, and think the best, is protargol. This I have given a careful and critical trial, and can say that it is more certain and lasting in its results than any of the others. With potassium permanganate you are apt to observe sudden and unexpected relapses due to the fact that the permanganate does not destroy the gonococci. Protargol does kill the gonococci, and in a shorter time than any other agent that can be used by irrigation or injection. In accute gonorrhea I have irrigated cases in which the inflammation was so acute that the patient could not use a hand injection. I employed in these cases a warm solution of protargol, one-quarter of one per cent. This solution has never failed to give relief.

CASE 1.—Mr. A., aged twenty-three; clerk; second attack of gonorrhea; inflammation acute; thick discharge of pus; acute pain on urinating. An irrigation of protargol ¼ per cent. solution was made every day, and the patient used during the interval a hand injection of a one per cent. solution. After nine irrigations the discharge became thin and watery, and stopped on the tenth day. The hand injection was used four times a day in conjunction with the irrigations.

CASE 2.—Joseph J., aged nineteen; clerk in a newspaper office; first attack of gonorrhea of five days duration; thick, creamy discharge. I gave him one irrigation, and ordered eight ounces of protargol solution for injections. I did not see him for two weeks, and when he returned the discharge was thin and watery. He had used the hand injection four times a day during his absence, and had also taken internally salol in ten-grain doses, three times daily. I increased the strength of the protargol solution to one per cent. He used injections four times a day for one more week, when the discharge ceased.

CASES 3, 4, 5.—In three cases of subacute gonorrhea I employed one-half per cent. solution of protargol made with hydrant water. Two of the cases returned after two weeks for a further supply of the solution. They said that the discharge had ceased after the first bottle of four ounces, but had recommenced. I requested them to let me know of the results. One wrote to me that the discharge failed to appear after the second bottle had been used. The other called for more injections; he had no discharge, but wanted to prevent any possible return. In the third case I never heard of the outcome.

In posterior urethritis I employed deep injections of a two per cent. solution of protargol by means of an Ultzmann-Keyes syringe, making one injection each day, and increasing the strength of the solution one-fourth per cent. at each injection. Under this treatment the most aggravating cases have been permanently cured. Time and patience are demanded, for it will occasionally take from three to four weeks, and sometimes longer, to obtain a cure. In these cases, I increased the strength of the solution from one to eight per cent., and gave from five to thirty deep injections. One case was cured with five injections of a five per cent. solution of protargol. The injections were first made every second day, and salol in five grain doses was administered four times daily. In another case I began with a one-half per cent. solution, and ran it up to eight per cent. before a cure was obtained. Twenty deep injections, one each day, were found necessary. In each of these cases the patients complained of a sharp smarting immediately after the injection, which passed off after five minutes. They then experienced quite some relief which lasted nearly all day.

In cystitis and irritation at the neck of the bladder the deep injection of a one per cent. protargol solution proved of decided benefit. Although at first followed by a sharp pain, a distinct sedative effect was produced, lasting on the average over ten hours, and in one instance over two days. I followed this method in the case of a butcher, who had three previous attacks of gonorrhea, the last one having never been cured. He complained of a continuous burning irritation in the posterior urethra and a slight discharge. I gave him a deep injection of a three per cent. solution of protargol, which caused considerable pain for about five minues, after which it subsided. He returned at the end of two days, and reported that he had felt comfortable all the time he was away, and as the burning had returned he came back for another injection. I gave him fourteen injections, one each day for one week, then one every second day. The discharge stopped, the burning ceased, and this result has still persisted.

In some cases, however, in which deep injections were not employed good results were obtained from the use of anterior injections as in the acute cases, as is shown by the two following histories:

CASE 6.—T. L., student; aged eighteen. This case was of eight months' standing. He had been through all kinds of treatment. There was a slight mucous discharge, but no pain nor burning. I prescribed a solution of protargol, one-half per cent., to use as an injection, four times a day. He returned in one week, stating that the discharge was less and only present in the morning. It must be mentioned that the patient persisted in riding a bicycle. He was told to continue the same treatment, but at his next visit, four days later, the discharge was unchanged, this being due to the fact that he had been drinking beer. I ordered a two per cent. solution of protargol in equal parts of glycerine and distilled water to be injected five times a day. On his return in five days the discharge was slight, and only found in the morning, and then only a drop or two. He was told to continue the treatment, but as I have not seen him since, he is either cured or has changed his doctor.

CASE 7.—J. F., aged twenty-five; mechanic; first attack, but of long standing; no pain; slight discharge. He had used almost all of the standard injections, from zinc sulphate up. I secured a specimen of his urine, and on allowing it to stand there formed a thick, tenacious mass, which in spite of any amount of shaking clung to the bottom of the bottle. I gave him four ounces of a one per cent. protargol solution prepared with hydrant water, and told him to inject five times a day, and retain the injection for five to ten minutes. He returned in four days for more injections, and brought another specimen of his urine, which contained considerable shreds and flakes, but on standing there was no tenacious deposit as before. The protargol solution of the same strength was continued, and at his next visit the urine was clear and free from shreds, and the discharge had stopped.

In hypertrophy of the prostate gland a one per cent. solution of protargol injected around the neck of the bladder will afford great relief, lessen the desire to urinate, and remove part of the irritation.

CASE 8.—Mr. T., aged seventy; retired merchant; had had cystitis for many years. Small calculi were present associated with hypertrophy of the prostate gland. He refused operation, and asked for relief. He complained of severe irritation and frequent urination, about every fifteen minutes, and then just a few drachms. A deep injection of a one per cent. solution of protargol, prepared from distilled water, was made. This gave him considerable relief, and reduced the frequency of urination; after the first injection he urinated once every three-quarters of an hour. This treatment is being pursued at the present time, and will probably have to be continued until the end of his life in order to render him more comfortable.

